# Supplementary material for: Micronutrient Status of Electronic Waste Recyclers at Agbogbloshie, Ghana
Source: Int J Environ Res Public Health. 2020 Dec 21;17(24):9575. doi: 10.3390/ijerph17249575 (PMC7767402; doi:10.3390/ijerph17249575)
Supplement: Supplementary file 1 [file ijerph-17-09575-s001.pdf]

Supplementary Materials

Table S1. Summary of elemental biomarker quality control measures.

Biomarker	Element	Detection Limit (μg/L)	Detection Frequency	Accuracy	Precision
Blood Elements	Ca	70,747	21%	N.A	10%
	Mg	1002.4	100%	N.A	8%
	Fe	2299.1	100%	N.A	7%
	Se	9.1	100%	86%	8%
	Cu	154.3	100%	103%	8%
	Zn	11,169.4	13%	94%	11%
Urinary Elements	Ca	7336.1	93%	N.A	9%
	Mg	347.6	100%	N.A	5%
	Fe	170.5	19%	N.A	19%
	Se	1.3	99%	63%	7%
	Cu	362.2	100%	109%	26%
	Zn	133.3	100%	123%	24%

NA—Not Available.

Table S2. Mean probability of dietary micronutrient adequacy (= % > RDA) of e-waste recyclers and controls.

Dietary Micronutrient Intake (mg)	E-Waste Recyclers n (%)	Controls n (%)	*p*-value
Ca	6 (6)	2 (3.9)	0.59
Mg	2 (2)	0 (0)	0.31
Se	9 (9)	11 (21.57)	0.03
Zn	46 (46)	15 (29.4)	0.05
Cu	10 (10)	3 (5.9)	0.39
Fe	96 (96)	50 (98.0)	0.51
